# The complete mitochondrial genomes of two band-winged grasshoppers, *Gastrimargus marmoratus *and *Oedaleus asiaticus*

**DOI:** 10.1186/1471-2164-10-156

**Published:** 2009-04-10

**Authors:** Chuan Ma, Chunxiang Liu, Pengcheng Yang, Le Kang

**Affiliations:** 1 State Key Laboratory of Integrated Management of Pest Insects and Rodents, Institute of Zoology, Chinese Academy of Sciences, Beijing 100101, PR China

## Abstract

**Background:**

The two closely related species of band-winged grasshoppers, *Gastrimargus marmoratus *and *Oedaleus asiaticus*, display significant differences in distribution, biological characteristics and habitat preferences. They are so similar to their respective congeneric species that it is difficult to differentiate them from other species within each genus. Hoppers of the two species have quite similar morphologies to that of *Locusta migratoria*, hence causing confusion in species identification. Thus we determined and compared the mitochondrial genomes of *G. marmoratus *and *O. asiaticus *to address these questions.

**Results:**

The complete mitochondrial genomes of *G. marmoratus *and *O. asiaticus *are 15,924 bp and 16,259 bp in size, respectively, with *O. asiaticus *being the largest among all known mitochondrial genomes in Orthoptera. Both mitochondrial genomes contain a standard set of 13 protein-coding genes, 22 transfer RNA genes, 2 ribosomal RNA genes and an A+T-rich region in the same order as those of the other analysed caeliferan species, but different from those of the ensiferan species by the rearrangement of *trnD *and *trnK*. The putative initiation codon for the *cox1 *gene in the two species is ATC. The presence of different sized tandem repeats in the A+T-rich region leads to size variation between their mitochondrial genomes. Except for *nad2*, *nad4L*, and *nad6*, most of the caeliferan mtDNA genes exhibit low levels of divergence. In phylogenetic analyses, the species from the suborder Caelifera form a monophyletic group, as is the case for the Ensifera. Furthermore, the two suborders cluster as sister groups, supporting the monophyly of Orthoptera.

**Conclusion:**

The mitochondrial genomes of both *G. marmoratus *and *O. asiaticus *harbor the typical 37 genes and an A+T-rich region, exhibiting similar characters to those of other grasshopper species. Characterization of the two mitochondrial genomes has enriched our knowledge on mitochondrial genomes of Orthoptera.

## Background

The band-winged grasshoppers, species of the subfamily Oedipodinae in Acridiidae, encompass over 900 described species. Indeed, the band-winged grasshoppers include some of the best known and most notorious pests in the world. Two species of band-winged grasshoppers, *Gastrimargus marmoratus *(Thunberg) and *Oedaleus asiaticus *Bei-Bienko, are listed as major pest Orthoptera species due to their damage to agriculture [1,2]. *G. marmoratus *is mainly distributed in the tropical and warm grassland ecosystems in South East Asia, as well as southern and east coastal China. In such areas *G. marmoratus *is able to move from the wild vegetation to farmlands with rice, maize, sugarcane, or other crops [3]. *O. asiaticus *is only distributed in the Mongolian Plateau and the Transbaikal region of the southern Russia [4]. It often reaches high population density in overgrazed steppes and xerophytous habitats [5]. *O. asiaticus *can form swarm bands and exhibit gregarious-like behaviors. In the steppe region of Inner Mongolia, it feeds mainly on grasses, such as *Leymus chinensis*, *Stipa *spp. and *Cleistogenes squarrosa *[6]. Although some biological and ecological research has been done on the two grasshopper species, studies concerning their nucleic and mitochondrial DNA sequences still remain scarce.

There are 23 species in *Gastrimargus *and 27 in *Oedaleus *in the world. Many of these are highly localized; however, *G. marmoratus *and *O. asiaticus *are widespread and abundant. The morphology of congeneric species in the two genera is so similar that it is difficult to differentiate one from another. Molecular phylogenetic studies have shown that the three genera *Gastrimargus, Oedaleus*, and *Locusta *are closely related [7]. The migratory locust, *Locusta migratoria*, a noxious pest insect in the world, is often found in sympatry with *G. marmoratus *or *O. asiaticus*, although the geographical ranges of the two latter species don't overlap. The morphological similarity among hoppers of the three species has sometimes caused confusion in the field. Recent concerns about species confusion raised during estimations of hopper density and dispersal for management practices emphasize the need for accurate nymphal identification. Therefore, the identification of highly polymorphic genetic markers such as mitochondrial sequences or nucleic microsatellites has been eagerly sought.

Mitochondrial genome sequences have been extensively used for inferring phylogenetic relationships. An accumulating body of evidence reveals that analyses based on whole mitochondrial sequence data yield trees with good resolution from higher-level groups down to closely related species [8,9]. Although the Orthoptera encompasses about 22,500 described species in the world [10], merely five complete mitochondrial genome sequences were available in the GenBank when we started this study (currently 12 species have been sequenced). The monophyly of Orthoptera has been widely accepted, and was supported by morphological [11] and molecular data [12]. However, the monophyly was not recovered in previous phylogenetic analyses based on mtDNA sequences [13,14]. Recently, a study using mitochondrial genome sequences confirmed the monophyly of Orthoptera [15]. One possible reason for such contrasting results may be insufficient taxon sampling, with only a single species representing each of the two Orthoptera suborders in earlier studies [13]. Thus, the addition of new complete mitochondrial genomes of orthopteran species will contribute to understanding of phylogenetic relationships in the Orthoptera and Insecta.

In this study, we determined the complete sequences of the mitochondrial genomes of *G. marmoratus *and *O. asiaticus*, and compared in detail the full sequences of both species. In addition, we analysed the phylogeny of 14 orthopteran species and 10 other polyneopteran species, as well as 4 outgroup insect species based on a concatenation of 13 mitochondrial protein-coding genes.

## Methods

### Samples and mitochondria isolation

Samples of *G. marmoratus *and *O. asiaticus *were collected in Hainan (109.47°E, 19.02°N) and Inner Mongolia (116.08°E, 43.94°N), China, respectively. They were preserved in 95% ethanol and maintained at 4°C. The mitochondria isolation for both species was performed according to Tamura and Aotsuka [16], with some modifications. A small portion of muscle tissue from a single individual was homogenized in 2 ml of chilled buffer (5 mM Tris, 70 mM sucrose, 220 mM Mannitol and 2 mM EDTA, pH 7.4). The homogenate was centrifuged at 500 g for 10 min at 4°C, and the supernatant was recovered and centrifuged at 800 g for 10 min at 4°C to pellet the nuclei and cellular debris. The resulting supernatant was centrifuged at 12,000 g for 10 min at 4°C to pellet the mitochondria.

### Mitochondrial DNA extraction

A modified method of salt-extraction protocol [17] was used to extract mtDNA from the isolated mitochondria. The mitochondria were resuspended in 330 μl of buffer (pH 8.2) containing 100 mM Tris, 40 mM NaCl and 2 mM EDTA. Then, 13 μl of 20% SDS and 6 μl of 20 mg/ml proteinase K were added to the mixture and incubated at 60°C for at least 2 hours or overnight, after which 250 μl of 5.3 M NaCl was added. The mixture was centrifuged at 5,000 rpm for 10 min at 4°C. The supernatant was transferred to a fresh tube, and 480 μl of chilled isopropanol was added and centrifuged at 12,000 g for 15 min at 4°C to pellet mtDNA. The pellet was washed with 75% ethanol, dried and then resuspended in sterile ddH2O.

### Genome determination and molecular analyses

Nine and seven pairs of PCR primers (See additional file 1: List of primers used for PCR amplification) were designed to amplify overlapping segments of the entire mitochondrial genomes of *G. marmoratus *and *O. asiaticus*, respectively. Two fragments of about 4.5 kb were amplified using LA Taq™ (Takara Biomedical, Japan) with an initial denaturation at 94°C for 2 min, followed by 30 cycles of denaturation at 94°C for 30 s, annealing at 55°C for 30 s, and extension at 72°C for 4.5 min, with a final elongation at 72°C for 7 min after the last cycle. The other fragments (~2 kb) were amplified using Takara EX Taq™ (Takara Biomedical, Japan) under the following conditions: 3 min at 94°C, followed by 35 cycles of 30 s at 94°C, 30 s at 50 – 55°C, 2 – 2.5 min at 72°C, and a final extension period of 7 min at 72°C. After purification with AxyPrep™ DNA Gel Extraction Kit, all PCR products were sequenced directly by means of primer walking. Sequencing was performed using BigDye terminator chemistry and ABI 3730x1 DNA Analyzer.

Sequence data were assembled using SeqMan software (DNAStar, Inc.). Transfer RNAs were identified by tRNAscan-SE 1.21 [18], and the other genes were determined by comparison with those of the sequenced orthopterans. Strand skew values were calculated according to the formulae by Perna et al. [19]. Divergences of *cox1 *sequences were calculated using MEGA 4.1 Beta [20] with the Kimura-2-Parameter model. The formula for calculating the divergence in the sliding-window analysis was as per Proutski et al. [21].

### Phylogenetic analyses

A total of 24 polyneopteran mitochondrial genomes available in the GenBank were included in our analyses (Table 1). For outgroups, we excluded the taxa that had been reported to introduce phylogenetic errors, based on the following criteria [14]: extreme compositional bias, inversion or translocation of genes on the opposite strand, inversion of the A+T-rich region, or lack of *atp8*. As a result, one species from Archaeognatha, one species from Zygentoma, and two species from Hemiptera were selected as outgroups. This selection appears appropriate considering both insect phylogeny and outgroup choice in previous studies [12,22].

**Table 1:** List of taxa used in the phylogenetic analysis.

Order	Family	Species	Accession number	Reference
Orthoptera	Acrididae	*Locusta migratoria*	NC_001712	[32]
Orthoptera	Acrididae	*Gastrimargus marmoratus*	EU513373	This study
Orthoptera	Acrididae	*Oedaleus asiaticus*	EU513374	This study
Orthoptera	Acrididae	*Oxya chinensis*	NC_010219	[33]
Orthoptera	Acrididae	*Acrida willemsei*	NC_011303	[15]
Orthoptera	Acrididae	*Calliptamus italicus*	NC_011305	[15]
Orthoptera	Acrididae	*Chorthippus chinensis*	NC_011095	[34]
Orthoptera	Gryllotalpidae	*Gryllotalpa orientalis*	NC_006678	[13]
Orthoptera	Gryllotalpidae	*Gryllotalpa pluvialis*	NC_011302	[15]
Orthoptera	Myrmecophilidae	*Myrmecophilus manni*	NC_011301	[15]
Orthoptera	Tettigoniidae	*Gampsocleis gratiosa*	NC_011200	[39]
Orthoptera	Tettigoniidae	*Anabrus simplex*	NC_009967	[40]
Orthoptera	Tettigoniidae	*Ruspolia dubia*	NC_009876	[41]
Orthoptera	Rhaphidophoridae	*Troglophilus neglectus*	NC_011306	[15]
Mantodea	Mantidae	*Tamolanica tamolana*	NC_007702	[53]
Blattaria	Blattidae	*Periplaneta fuliginosa*	NC_006076	[54]
Isoptera	Rhinotermitidae	*Reticulitermes flavipes*	NC_009498	[55]
Isoptera	Rhinotermitidae	*Reticulitermes hageni*	NC_009501	[55]
Isoptera	Rhinotermitidae	*Reticulitermes santonensis*	NC_009499	[55]
Isoptera	Rhinotermitidae	*Reticulitermes virginicus*	NC_009500	[55]
Mantophasmatodea	Mantophasmatidae	*Sclerophasma paresisense*	NC_007701	[53]
Phasmatodea	Timematidae	*Timema californicum*	DQ241799	[53]
Grylloblattodea	Grylloblattidae	*Grylloblatta sculleni*	DQ241796	[53]
Plecoptera	Pteronarcyidae	*Pteronarcys princeps*	NC_006133	[56]
Hemiptera	Aphrophoridae	*Philaenus spumarius*	NC_005944	[49]
Hemiptera	Reduviidae	*Triatoma dimidiata*	NC_002609	[50]
Zygentoma	Lepidotrichidae	*Tricholepidion gertschi*	NC_005437	[9]
Archaeognatha	Meinertellidae	*Nesomachilis australica*	NC_006895	[51]

The nucleotide and amino acid sequences of the protein-coding genes were retrieved from the Mitome database [23]. The amino acid sequences were individually aligned using BioEdit [24], followed by manual refinements. The corresponding nucleotide sequences were retro-aligned, using the PAL2NAL webserver [25], and then concatenated into a single alignment. With "codons" selected as the type of sequence and other default options, the program Gblocks [26] was applied to remove poorly aligned positions of the nucleotide alignment. Third codon positions were highly saturated as was determined by DAMBE [27]. To investigate the effect of mutational saturation, we employed phylogenetic analyses based on the two data sets: (i) DNA alignment including only first and second codon positions, and (ii) DNA alignment with all codon positions included. The TVM+I+G model was selected as the best-fit one by the program ModelTest (ver. 3.7) [28] based on the Akaike Information Criterion.

Bayesian Inference (BI) and Maximum Likelihood (ML) methods were employed to analyze the two data sets. BI analysis was performed using MrBayes, ver.3.1.2 [29]. Two sets of four chains were allowed to run simultaneously for 1,000,000 generations. Each set was sampled every 100 generations with a burnin of 25%. Stationarity was considered to be reached when the average standard deviation of split frequencies was less than 0.01. Bayesian posterior probabilities (BPP) were estimated on a 50% majority rule consensus tree of the remaining trees. ML analysis was conducted using the program TreeFinder [30] with GTR substitution model. Bootstrap analysis was performed with 500 replicates.

## Results and discussion

### General features

The complete mtDNA sequences of *G. marmoratus *and *O. asiaticus *are 15,924 bp and 16,259 bp in size, respectively (Table 2). The two mitochondrial genomes have been deposited in the GenBank database under the accession numbers EU513373 (for *G. marmoratus*) and EU513374 (for *O. asiaticus*). The mitochondrial genome sequence of *O. asiaticus *is the longest in all orthopteran mitochondrial genomes available in the GenBank. Its relatively large size is mainly owing to the extended A+T-rich region caused by the presence of tandem repeats. Both mitochondrial genomes share the same 37 typical metazoan genes (13 protein-coding genes, 22 transfer RNA genes, and 2 ribosomal RNA genes) and an A+T-rich region [31], and they have identical gene arrangement with *L. migratoria *[32],*Oxya chinensis *[33], *Chorthippus chinensis *[34], *Calliptamus italicus *[15], and *Acrida willemsei *[15]. In addition to the A+T-rich region, 72 and 98 noncoding nucleotides are present in the mitochondrial genomes of *G. marmoratus *and *O. asiaticus*, respectively. There are overlapping genes in both mitochondrial genomes as in other metazoan mitochondrial genomes. In *G. marmoratus*, the overlaps occur six times and involve a total of 35 bp, lacking only one 1-bp overlap between *trnQ *and *trnM *compared with *O. asiaticus*. These overlaps exist between *atp8 *and *atp6 *on the majority strand, *nad4L *and *nad4 *on the minority strand, and between some adjacent genes oriented on opposite strands.

**Table 2:** Annotation of the mitochondrial genomes of *Gastrimargus marmoratus *(*Gm*) and *Oedaleus asiaticus *(*Oa*).

Feature	Strand	Position		Initiation codon/Stop codon		Anticodon
				
		*Gm*	*Oa*	*Gm*	*Oa*	
*trnI*	J	1–65	1–65			GAT
*trnQ*	N	63–131	63–131			TTG
*trnM*	J	135–203	131–199			CAT
*nad2*	J	204–1224	200–1220	ATG/T--	ATG/T--	
*trnW*	J	1225–1290	1221–1286			TCA
*trnC*	N	1283–1351	1279–1344			GCA
*trnY*	N	1362–1429	1354–1420			GTA
*cox1*	J	1422–2961	1413–2952	ATC/T--	ATC/T--	
*trn L(UUR)*	J	2962–3027	2953–3018			TAA
*cox2*	J	3031–3712	3027–3708	ATG/T--	ATG/T--	
*trnD*	J	3713–3776	3709–3772			GTC
*trnK*	J	3780–3850	3776–3846			CTT
*atp8*	J	3868–4026	3864–4022	ATC/TAA	ATC/TAA	
*atp6*	J	4020–4697	4016–4693	ATG/TAA	ATG/TAA	
*cox3*	J	4702–5493	4698–5489	ATG/TAA	ATG/TAA	
*trnG*	J	5496–5560	5492–5555			TCC
*nad3*	J	5561–5912	5556–5907	ATT/T--	ATT/T--	
*trnA*	J	5913–5977	5908–5972			TGC
*trnR*	J	5981–6044	5977–6043			TCG
*trnN*	J	6045–6111	6044–6111			GTT
*trnS (AGN)*	J	6112–6178	6112–6178			GCT
*trnE*	J	6179–6245	6179–6244			TTC
*trnF*	N	6244–6308	6243–6308			GAA
*nad5*	N	6309–8028	6309–8025	ATT/T--	ATT/T--	
*trnH*	N	8044–8109	8041–8108			GTG
*nad4*	N	8111–9445	8110–9444	ATG/TAG	ATG/TAA	
*nad4L*	N	9439–9732	9438–9731	ATG/TAA	ATG/TAA	
*trnT*	J	9735–9798	9734–9797			TGT
*trnP*	N	9799–9863	9798–9862			TGG
*nad6*	J	9866–10390	9865–10386	ATG/TAA	ATG/TAA	
*cob*	J	10395–11533	10394–11532	ATG/TA-	ATG/TA-	
*trnS (UCN)*	J	11534–11603	11533–11603			TGA
*nad1*	N	11604–12569	11625–12569	ATG/TAA	ATA/TAA	
*trnL (CUN)*	N	12573–12638	12573–12638			TAG
*rrnL*	N	12639–13960	12639–13956			
*trnV*	N	13961–14032	13957–14027			TAC
*rrnS*	N	14033–14863	14028–14858			
A+T-rich region	J	14864–15924	14859–16259			

J and N refer to the majority and minority strand, respectively. Position numbers refer to positions on the majority strand.

The nucleotide compositions of the entire mtDNA sequences for *G. marmoratus *and *O. asiaticus *are significantly biased toward A and T. The A+T content is 75.18% (A = 45.57%, T = 29.62%, C = 15.22%, G = 9.60%) in *G. marmoratus *and 75.39% in *O. asiaticus *(A = 45.03%, T = 30.36%, C = 14.57%, G = 10.04%; see Table 3). On the other hand, both of the majority strands of *G. marmoratus *(AT-skew = 0.212, GC-skew = -0.226) and *O. asiaticus *(AT-skew = 0.195, GC-skew = -0.184) favor A and C. In mammals, the underlying mechanism for this bias of strand-specific nucleotide composition is the deamination of C and A in the H strand during replication [35].

**Table 3:** Nucleotide compositions of *Gastrimargus marmoratus *(*Gm*) and *Oedaleus asiaticus *(*Oa*).

Feature	A (%)		C (%)		G (%)		T (%)		A+T (%)	
					
	*Gm*	*Oa*	*Gm*	*Oa*	*Gm*	*Oa*	*Gm*	*Oa*	*Gm*	*Oa*
Whole genome	45.57	45.03	15.22	14.57	9.60	10.04	29.62	30.36	75.18	75.39
Protein-coding genes*	33.31	33.32	13.66	13.61	12.43	12.53	40.60	40.55	73.91	73.86
1st codon positions	34.50	34.23	12.32	12.54	18.39	18.81	34.79	34.42	69.29	68.65
2nd codon positions	20.19	19.91	19.89	20.16	14.20	14.21	45.72	45.72	65.91	65.64
3rd codon positions	45.23	45.80	8.78	8.13	4.70	4.57	41.29	41.50	86.52	87.30
tRNA genes	38.50	38.21	10.95	11.01	14.22	14.34	36.33	36.44	74.83	74.64
*rrnL *genes	32.45	32.09	7.87	7.74	13.99	14.11	45.69	46.05	78.14	78.15
*rrnS *genes	29.96	29.48	8.78	9.51	15.52	15.04	45.73	45.97	75.69	75.45
A+T-rich region	51.93	48.89	10.37	8.78	5.37	6.71	32.33	35.62	84.26	84.51

* Stop codons were excluded.

### Transfer and ribosomal RNA genes

The complete set of 22 tRNA genes typical of metazoan mitochondrial genomes is present in the two mitochondrial genomes: two for serine and leucine, and one for the other amino acids. All tRNA genes were determined by tRNAscan-SE 1.21 [18], except for *trnH *in *G. marmoratus *and *trnS(AGN) *in both mitochondrial genomes, which were determined through sequence comparison with previously published orthopteran mitochondrial genomes. Twenty-one tRNA genes can be folded into the typical cloverleaf structure, whereas *trnS(AGN) *in both mitochondrial genomes has an unpaired stretch of 11 nucleotides instead of the DHU arm, as is often found in arthropod mitochondrial genomes. Both mitochondrial genomes have the identical nucleotide sequence for only *trnL(CUN)*. Unmatched base pairs have been observed in stems of tRNA secondary structures. In *G. marmoratus*, there are 22 unmatched base pairs, consisting of 19 G-U pairs, 1 A-A and 2 U-U mismatches; whereas in the case of *O. asiaticus*, 16 G-U pairs, 1 A-A and 3 U-U mismatches have been identified. Their anticodons are identical to those of all the other available orthopteran mitochondrial genomes. Compared with the published ensiferan mitochondrial gene order *cox2*-*trnK*-*trnD*-*atp8*, there is a rearrangement of *trnD *and *trnK *in both *G. marmoratus *and *O. asiaticus *mitochondrial genomes, as well as in those of all the other caeliferans determined so far. This is consistent with the previous finding that in the Orthoptera such rearrangement only occurs within the suborder Caelifera [36].

The two genes encoding the large and small ribosomal RNA subunits (*rrnL *and *rrnS*) are located between *trnL(CUN) *and *trnV*, and between *trnV *and the A+T-rich region, respectively. The length of *rrnL *is 1,322 bp in *G. marmoratus *and 1,318 bp in *O. asiaticus*, with an A+T content of 78.14% and 78.15%, respectively. The *rrnS *is 831 bp in both mitochondrial genomes, and the A+T content is 75.69% for *G. marmoratus *and 75.45% for *O. asiaticus *(Table 3).

### Protein-coding genes

**Figure 1:**
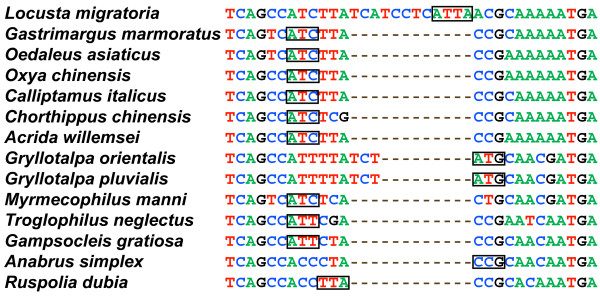
**The alignment of the *cox1 *start region of orthopteran mitochondrial genomes currently available**. Hyphens indicate inferred gaps. Boxed nucleotides have been proposed to act as initiation codons, except for ATC in *G. marmoratus*, *O. asiaticus *and *A. willemsei*, which was proposed in our analysis.

All of the 13 *G. marmoratus *protein-coding genes start with a typical ATN codon: two (*nad3*, *nad5*) with ATT, two (*cox1*, *atp8*) with ATC and the other nine with ATG (Table 2). In comparison with *G. marmoratus*, merely *nad1 *in *O. asiaticus *possesses a different initiation codon ATA. Previous studies reported no typical ATN initiation codon for *cox1 *in mitochondrial genomes of many species. As a consequence, many other irregular initiation codons, such as ATTA [37] and ATTTAA [38], were postulated. In *G. marmoratus *and *O. asiaticus*, however, the putative initiation codon for *cox1 *is ATC, which is located 8-bp upstream of the adjacent *trnY*. In the *cox1 *start region, *L. migratoria *has an insert of 9 or 12 nucleotides compared with the other orthopterans (Figure 1). The initiation codon for *cox1 *remains ambiguous in *L. migratoria*, where a 4-bp start sequence, ATTA, has been proposed [32]. The counterparts for *O. chinensis *[33], *C. italicus *[15], and *C. chinensis *[34] have been presumed to be ATC. Based on the *cox1 *sequence in *A. willemsei *(GenBank: NC_011303), ATC could be regarded as the potential initiation codon. Therefore, the caeliferan species share the same initiation codon ATC except for *L. migratoria*. In both mole crickets *Gryllotalpa orientalis *[13] and *Gryllotalpa pluvialis *[15], *cox1 *has a canonical initiation codon ATG. The initiation codon is ATC in the tettigoniid species like *Myrmecophilus manni *[15] and ATT in both *Troglophilus neglectus *[15] and *Gampsocleis gratiosa *[39]. The triplet CCG has been identified as the *cox1 *initiation codon for the cricket *Anabrus simplex *[40]. Although the corresponding triplet is also CCG for the katydid *Ruspolia dubia*, TTA has been proposed to initiate *cox1 *[41]. Annotation of a new mitochondrial genome is commonly carried out by comparison with closely related mitochondrial genomes already determined. This approach, therefore, has limitations to this extent. The precise initiation codon for *cox1 *can be finally determined by protein sequencing.

Seven of the 13 protein-coding genes terminate with the conventional stop codons TAG or TAA, and the remaining ones have incomplete stop codons T or TA adjacent to a downstream tRNA gene (Table 2). The only difference in stop codons between the two mitochondrial genomes lies in that *nad4 *gene in *G. marmoratus *uses TAG, while the stop codon is TAA in *O. asiaticus*. The secondary structure of tRNA genes facilitates correct processing of the polycistronic transcript into mature RNA molecules [42]. The presence of incomplete stop codon is common in metazoan mitochondrial genomes, and these truncated stop codons are presumed to be completed via post-transcriptional polyadenylation [42]. In accordance with other arthropods, overlapping protein-coding genes are present in both *G. marmoratus *and *O. asiaticus *mitochondrial genomes; a 7-bp overlap exists not only between *atp8 *and *atp6 *but also between *nad4L *and *nad4*. In this case, hairpin structures forming at the 3' end of the upstream protein's mRNA, rather than secondary structures of tRNA genes, may act as a signal for the cleavage of the polycistronic primary transcript [40].

A DNA barcoding approach based on *cox1 *sequence diversity has been utilized for identification of closely allied species [43]. We calculated pairwise divergences of a 650-bp sequence (corresponding to nucleotide positions 1428–2077 in *G. marmoratus *mitochondrial genome) from the *cox1 *5' terminus in the fourteen known orthopteran mitochondrial genomes (See additional file 2: Pairwise divergences (%) of a 650-bp sequence from cox1 5' terminus). Pairwise between the caeliferans and the ensiferans exhibits overall high divergences. The pairwise divergences range from 11.04% to 31.65%, indicating that this fragment of *cox1 *gene is effective enough to discriminate these species. However, whether the *cox1 *barcode sequence could be applied to the whole Orthoptera requires broad taxon sampling.

The nucleotide sequence identities of *G. marmoratus *and *O. asiaticus *protein-coding genes range from 84.3% (*nad6*) to 92.1% (*nad4L*; see Table 4). Based on identity of inferred amino acid sequences, *cox1 *(97.0%) is the most conserved protein-coding gene, while *atp8 *(80.7%) is the least conserved with a variable domain at the C-terminus.

**Table 4:** Sequence identity of *G. marmoratus *and *O. asiaticus *protein-coding genes.

gene	number of codons		% sequence identity	
		
	*G. marmoratus*	*O. asiaticus*	nucleotide	Amino acid
*atp6*	225	225	88.9	92.8
*atp8*	52	52	86.1	80.7
*cox1*	513	513	90.1	97.0
*cox2*	227	227	91.2	95.1
*cox3*	263	263	89.3	93.1
*cob*	379	379	87.5	91.5
*nad1*	321	314	90.4	92.8
*nad2*	340	340	86.0	84.4
*nad3*	117	117	89.4	89.7
*nad4*	444	444	89.8	91.2
*nad4L*	97	97	92.1	95.8
*nad5*	573	572	91.3	92.4
*nad6*	174	173	84.3	83.9

The A+T content of protein-coding genes, excluding stop codons, is 73.91% and 73.86% in *G. marmoratus *and *O. asiaticus*, respectively (Table 3). This significant AT-bias affects codon usage in proteins, with ATT (encoding isoleucine) being the most frequently used codon and GC-rich codons being least frequently used (e.g., CGC is absent in both mitochondrial genomes). In *G. marmoratus *and *O. asiaticus*, when first, second and third codon positions are considered separately, the highest A+T content is in third codon positions (86.52% and 87.30%, respectively), the strongest bias toward T is in second codon positions (both 45.72%), and the lowest content of G is in third codon positions (4.70% and 4.57%, respectively; see Table 3).

### The A+T-rich region

This region has an A+T content of 84.26% in *G. marmoratus *and 84.51% in *O. asiaticus *(Table 3). The A+T-rich regions of two grasshopper species, *Schistocerca gregaria *and *Chorthippus parallelus*, have been sequenced [44]. The A+T-rich region, usually the largest noncoding part of the metazoan mitochondrial DNA molecule, evolves relatively fast due to few selective constraints. For the orthopterans, a high mutation rate of this region results in significant size variation ranging from 70 bp in the katydid *R. dubia *[41] to 1,512 bp in the grasshopper *C. parallelus *[44]. This variation is predominantly due to both length variation within tandem repeats and differences in their copy numbers.

In *G. marmoratus*, there are three tandem repeat units. The first one begins in the *rrnS *gene and extends into the A+T-rich region. The first two repeat units are 166 bp long and identical in sequence, slightly different from the third one (155 bp). The A+T content is 79.88% for these repeat sequences and 87.68% for the A+T-rich region excluding these repeat sequences. The longest open reading frame (255 bp in length, encoding 85 amino acids) detected in the A+T-rich region is located in the minority strand of these tandem repeats, but a tblastn research found no significant similarity with sequences in the GenBank database, suggesting it is a non-functional ORF. In *O. asiaticus*, the A+T-rich region contains two repeat regions. The first one (75.83% A+T) consists of two complete repeat units (155 bp) and one truncated repeat unit (141 bp), with 5 to 7 nucleotide substitutions between them. Of these repeat units, the first one is partially located in the *rrnS *gene. The minority strand of this repeat region has the longest open reading frame (237 bp) of the A+T-rich region, but we got negative results using the tblastn research. The other repeat region (90.35% A+T), situated near the *trnI *gene, comprises two repeat units (345 and 339 bp, respectively) with the shorter one truncated at 3' end. Similar large tandem repeats are also present in the A+T-rich region of *L. migratoria*, *C. parallelus*, *T. neglectus *and *G. gratiosa *but they are absent in the other orthopteran mitochondrial genomes determined so far. The fact that tandem repeats are non-conserved among these orthopteran mitochondrial genomes indicates a lack of a functional role for these tandem repeats. Replication slippage is regarded as a dominant mechanism to account for the existence of tandem repeats [45,46].

The A+T-rich region contains control elements for replication and transcription of animal mitochondrial genomes [31]. Of them, the stem-loop secondary structure is potentially involved in initiation of a second-strand replication [44]. The possible stem-loop structure located immediately upstream of the origin of the minority strand has been detected in *L. migratoria *[47]. Such stem-loop structures also exist in all the other previously determined caeliferan mitochondrial genomes. For *G. marmoratus *and *O. asiaticus*, using the program mfold [48], we have predicted the possible stem-loop structures, both of which have the same secondary structure and nucleotide sequence as that of *L. migratoria*. The conserved stem-loop structures in these mitochondrial genomes suggest their functional importance and may provide clues for understanding the initiation process of mtDNA replication.

### Divergence of mtDNA sequences

**Figure 2:**
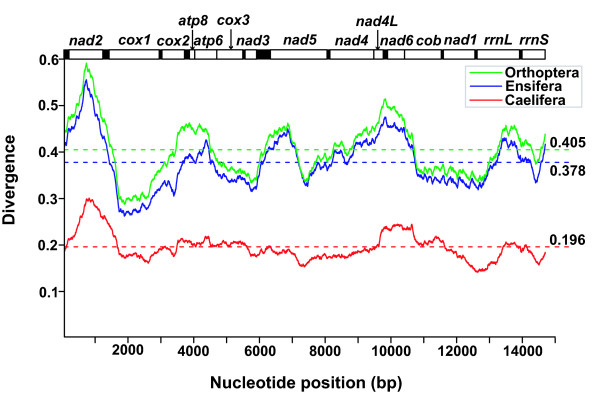
**Plot of divergences among the Orthoptera mtDNA sequences excluding the A+T-rich region**. The bar at the top illustrates the position of protein-coding genes and rRNAs, and the tRNAs are represented as black boxes. Dashed lines indicate mean divergence. The window is 1,000 bp in length and slides 1 bp at a time. The sliding-window analysis calculates the divergence of the 14 orthopterans, the 7 caeliferans, and the 7 ensiferans, respectively.

A sliding-window analysis was performed to compute divergence of the fourteen orthopteran mtDNA sequences excluding the A+T-rich region (Figure 2). The mean divergence of the fourteen orthopteran mtDNA sequences is 0.405. The *nad2 *gene has undergone accelerated evolution, as evidenced by the highest level of divergence. The *cox1 *gene is the most conserved protein-coding gene, and is therefore a useful marker for investigating phylogenetic relationships at higher taxonomic levels. Divergence of mtDNA sequences of the seven caeliferans and the seven ensiferans was also calculated, respectively (Figure 2). The seven ensiferans has not only overall similar sequence divergence pattern to that of the fourteen orthopterans but much higher divergence (mean divergence = 0.378) than the seven caeliferans (mean divergence = 0.196), indicating that the high divergence of the fourteen orthopterans attributes to the ensiferan mtDNA sequence divergence. By contrast, mtDNA sequences of the caeliferans are more conserved, except for sequences of *nad2*, *nad4L*, and *nad6*. Due to the highest divergence, the *nad2 *nucleotide sequence can be used as an effective molecular marker to analyse intraspecific relationships and to distinguish closely related grasshopper species.

### Phylogenetic relationships

**Figure 3:**
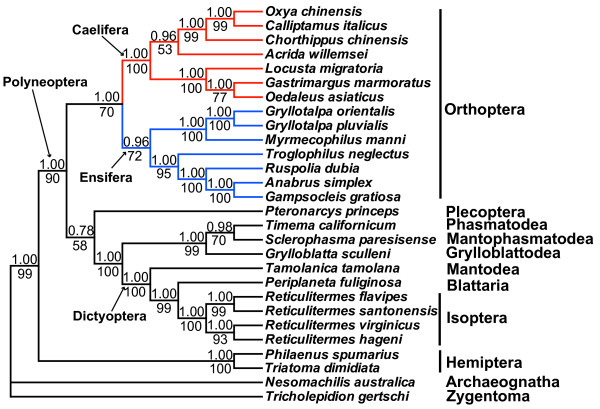
**Phylogenetic tree of 24 polyneopterans**. Phylogenetic analysis was based on first and second codon positions of 13 protein-coding genes. The tree was rooted by Archaeognatha, Zygentoma, and Hemiptera. Numbers refer to Bayesian posterior probabilities (BPP; above nodes) and bootstrap support values (BS; below nodes).

We performed phylogenetic analysis with nucleotide sequences of 13 mitochondrial protein-coding genes from 24 polyneopteran species and 4 outgroup insect species (*Philaenus spumarius *[49],*Triatoma dimidiate *[50],*Tricholepidion gertschi *[9] and *Nesomachilis australica *[51]). BI and ML analyses using only first and second codon positions of the 13 protein-coding genes generate identical tree topologies (Figure 3).

Polyneoptera refers to an assemblage of eleven insect orders, and its monophyly remains contentious [22]. Using both morphological and molecular data, Wheeler et al. [52] recovered monophyletic Polyneoptera. However, Terry & Whiting [22] supported the paraphyly of Polyneoptera based on an extensive data set (18S rDNA, 28S rDNA, Histone 3 DNA sequences, and 125 morphological characters). In the present study, eight orders of the polyneopteran lineages are included and they cluster as a monophyletic clade (Figure 3). Our result may provide evidence for resolving phylogenetic relationships of Polyneoptera, although the currently limited taxon sampling highlights the preliminary nature of this analysis. The unsampled Dermaptera, Embiidina, and Zoraptera, should be included in further studies to provide a more accurate phylogenetic estimate.

Dictyoptera (Isoptera, Blattaria, and Mantodea) is recovered as monophyletic (Figure 3), with *Tamolanica tamolana *[53] (Mantodea) as sister taxon to *Periplaneta fuliginosa *[54] (Blattaria) and four *Reticulitermes *species [55] (Isoptera). The relationships within Dictyoptera are in agreement with previous analyses [22]. The sister relationship between Mantophasmatodea and Phasmatodea is supported and this clade is sister to Grylloblattodea (Figure 3). Our result is consistent with previous analyses using mitochondrial protein-coding genes [14,53], but different from the study by Terry & Whiting [22] who suggested a sister-group relationship between Mantophasmatodea and Grylloblattodea.

Plecoptera, here represented by *Pteronarcys princeps *[56], is sister group to the assemblage (((Mantophasmatodea + Phasmatodea) + Grylloblattodea) + Dictyoptera), but the low support values (BPP = 0.78, BS = 58%; see Figure 3) suggest that the position of Plecoptera is not well resolved in the phylogenetic analysis. Plecoptera has been proposed as the sister taxon to Dermaptera and Zoraptera [22], whose mitochondrial genomes are not available. Carapelli et al. [14] clustered *P. princeps *with Diptera (flies) rather than polyneopterans, and detected no problematic characteristics within its mitochondrial genome. Later, when examining the phylogenetic signal from mitochondrial genome data, Fenn et al. [15] found that *P. princeps *introduced instability in phylogenetic analysis possibly due to base composition heterogeneity, and thus they excluded it. To test the effect caused by *P. princeps*, in the present study we have reconstructed the phylogeny by removing *P. princeps *and compared it with the phylogeny including this taxon. We find that exclusion of *P. princeps *makes no difference in placement of all other taxa but results in a more stable tree topology, as is evident from higher nodal supports (See additional file 3: Phylogenetic tree of Polyneoptera without *Pteronarcys princeps*). Although *P. princeps *has not considerably affected phylogenetic inference in our study, sampling other closely related plecopterans might be a strategy for further progress in the reconstruction of Polyneoptera phylogeny.

The two Orthoptera suborders, Caelifera and Ensifera, are both recovered as monophyletic (the latter taxon with a lower statistical support: BPP = 0.96, BS = 72%; see Figure 3). This result is consistent with traditional morphological taxonomics and previous studies [57,58]. The sister taxon relationship (BPP = 1.00, BS = 70%) between Caelifera and Ensifera supports the monophyly of Orthoptera [10-12,15]. Within Caelifera, five subfamilies of Acrididae are represented and Oedipodinae occupies the basal position. The ensiferan species split into two clades, (Tettigoniidae + Rhaphidophoridae) and (Gryllotalpidae + Myrmecophilidae), concordant with the study of Fenn et al. [15]. In contrast with the failure to recover the monophyletic Orthoptera in previous mtDNA-based phylogenetic analyses [13,14], our study demonstrates that analyses with only two species representing the Orthoptera may lead to false phylogenetic inferences. Nevertheless, given the more than 20,000 species of the Orthoptera, the present taxon sampling is still far from enough. To further clarify the phylogeny of the Orthoptera, more extensive sequencing of orthopteran mitochondrial genomes is required.

Our initial analysis using all codon positions of protein-coding genes leads to quite different tree topologies, compared with the tree based on only first and second codon positions. Neither the ML nor the BI tree recovers monophyletic Orthoptera (See additional file 4: Phylogenetic trees of Polyneoptera using all codon positions of 13 protein-coding genes). Furthermore, the assemblage (((Mantophasmatodea + Phasmatodea) + Grylloblattodea) + Dictyoptera) is sister either to Caelifera (BS = 53%) in the ML tree or to the clade of Ensifera and Plecoptera (BPP = 0.54) in the BI tree. It is likely due to the mutational saturation of third codon positions plaguing the phylogenetic analysis and subsequently decreasing support values. Therefore, the analysis using all codon positions is not suitable in the context of our taxon sampling, although Fenn et al. [15] found that inclusion of third codon positions did not negatively affect phylogenetic inference. Here, we regard the analysis using only first and second positions as our best estimate.

## Conclusion

The mitochondrial genomes of *G. marmoratus *and *O. asiaticus *have overall similarities. Both species, with other grasshopper species, share the same mitochondrial genome organization, which differs from that of the available ensiferan species by the translocation between *trnD *and *trnK*. The potential initiation codon for *cox1 *gene in *G. marmoratus *and *O. asiaticus *is ATC. In addition to stem-loop structures in the A+T-rich region, another common feature of both mitochondrial genomes is the existence of tandem repeats, but the kinds of repeats and the copy number of each repeat unit are variable. The sliding-window analysis reveals that mtDNA sequences of the analysed caeliferans have lower divergence than those of the ensiferans. The *nad2 *nucleotide sequence may serve as an effective marker to determine phylogenetic relationships of intraspecies and closely related grasshopper species. The phylogenetic analysis based on mtDNA sequences of 13 protein-coding genes confirms the monophyly of Orthoptera. The analyses of *G. marmoratus *and *O. asiaticus *mitochondrial genomes have added to our knowledge on mitochondrial genomes of Orthoptera.

## Abbreviations

*atp6 *and *atp8*: ATP synthase subunits 6 and 8; *cob*: cytochrome b; *cox1*-*3*: cytochrome c oxidase subunits 1–3; *nad1–6 *and *nad4L*: NADH dehydrogenase subunits 1–6 and 4L; *rrnS *and *rrnL*: small and large ribosomal RNA (rRNA) subunits; *trnX*: transfer RNA (tRNA) genes, where X is the one-letter abbreviation of the corresponding amino acid; BS: bootstrap support.

## Authors' contributions

LK was primarily responsible for the design, coordination and conduction of this study. CM was responsible for determining and assembling the mtDNA sequences, and drafted the manuscript, tables, and figures. CM and CL performed phylogenetic analyses. PY calculated divergence of mtDNA sequences. LK extensively revised the manuscript. All authors read and approved the final manuscript.

## Supplementary Material

Additional file 1 **List of primers used for PCR amplification**. * Numbers refer to the nucleotide positions of primers' 5 prime ends. Primers in bold were used to amplify ~4.5 Kb fragments with LA Taq™ polymerase. Click here for file

Additional file 2 **Pairwise divergences (%) of a 650-bp sequence from *cox1 *5' terminus**. Divergences were calculated using MEGA 4.1 Beta [20] with the Kimura 2-Parameter model. Click here for file

Additional file 3 **Phylogenetic tree of Polyneoptera without *Pteronarcys princeps***. Phylogenetic analysis was based on first and second codon positions of 13 protein-coding genes. To avoid the potential effect introduced by *P. princeps*, we excluded it in this analysis. Numbers refer to Bayesian posterior probabilities (above nodes) and bootstrap support values (below nodes). Click here for file

Additional file 4 **Phylogenetic trees of Polyneoptera using all codon positions of 13 protein-coding genes**. Numbers at nodes refer to Bayesian posterior probabilities (left tree) and ML bootstrap support values (right tree). Click here for file
