# Supplementary figures and images for: The complete mitochondrial genomes of two band-winged grasshoppers, Gastrimargus marmoratus and Oedaleus asiaticus
Source: BMC Genomics. 2009 Apr 10;10:156. doi: 10.1186/1471-2164-10-156 (PMC2674460; doi:10.1186/1471-2164-10-156)

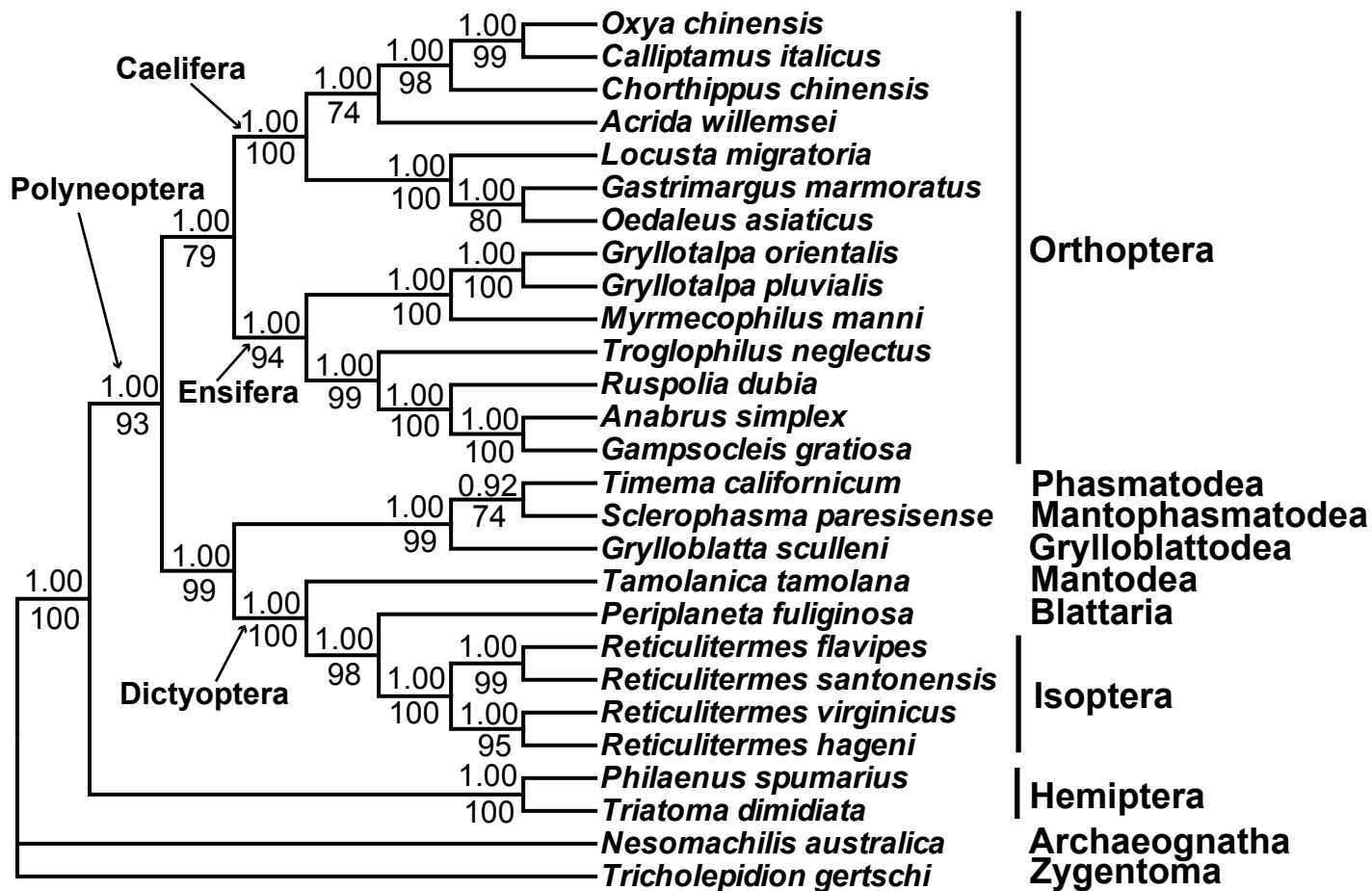

Supplement: Additional file 3 — Phylogenetic tree of Polyneoptera without Pteronarcys princeps. Phylogenetic analysis was based on first and second codon positions of 13 protein-coding genes. To avoid the potential effect introduced by P. princeps, we excluded it in this analysis. Numbers refer to Bayesian posterior probabilities (above nodes) and bootstrap support values (below nodes). [file 1471-2164-10-156-S3.pdf]
