# Supplementary material for: The complete mitochondrial genomes of two band-winged grasshoppers, Gastrimargus marmoratus and Oedaleus asiaticus
Source: BMC Genomics. 2009 Apr 10;10:156. doi: 10.1186/1471-2164-10-156 (PMC2674460; doi:10.1186/1471-2164-10-156)
Supplement: Additional file 4 — Phylogenetic trees of Polyneoptera using all codon positions of 13 protein-coding genes. Numbers at nodes refer to Bayesian posterior probabilities (left tree) and ML bootstrap support values (right tree). [file 1471-2164-10-156-S4.pdf]

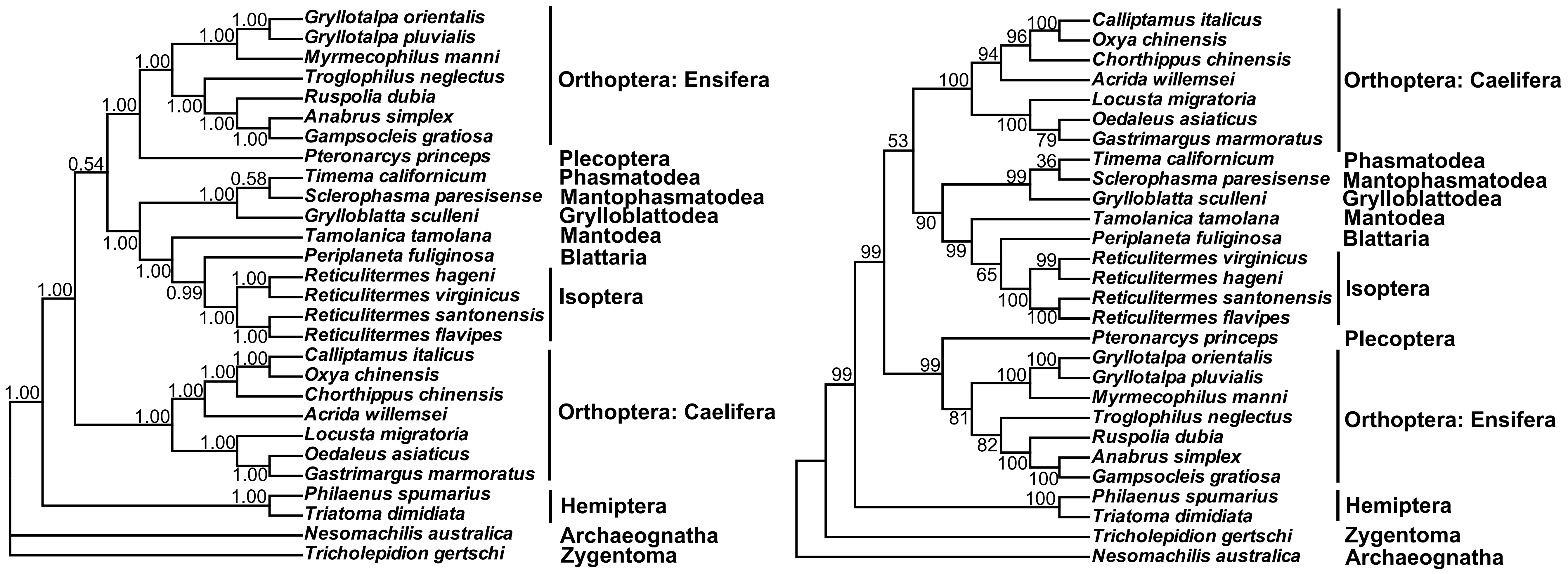
Gryllotalpa orientalis
Gryllotalpa pluvialis
Myrmecophilus manni
Troglophilus neglectus
Ruspolia dubia
Anabrus simplex
Gampsocleis gratiosa
Orthoptera: Ensifera
Pteronarcys princeps
Plecoptera
Timema californicum
Phasmatodea
Sclerophasma paresisense
Mantophasmatodea
Grylloblatta sculleni
Grylloblattodea
Tamolanica tamolana
Mantodea
Periplaneta fuliginosa
Blattaria
Reticulitermes hageni
Reticulitermes virginicus
Reticulitermes santonensis
Reticulitermes flavipes
Isoptera
Calliptamus italicus
Oxya chinensis
Chorthippus chinensis
Acrida willemsei
Locusta migratoria
Oedaleus asiaticus
Gastrimargus marmoratus
Orthoptera: Caelifera
Philaenus spumarius
Triatoma dimidiata
Hemiptera
Nesomachilis australica
Archaeognatha
Tricholepidion gertschi
Zygentoma
1.00
0.54
0.58
0.99
100
96
94
53
90
99
36
65
79
81
82
